# Desktop 3D printed anatomic models for minimally invasive direct coronary artery bypass

**DOI:** 10.1186/s41205-024-00222-1

**Published:** 2024-06-12

**Authors:** Prashanth Ravi, Michael B. Burch, Andreas A. Giannopoulos, Isabella Liu, Shayne Kondor, Leonid L. Chepelev, Tommaso H. Danesi, Frank J. Rybicki, Antonio Panza

**Affiliations:** 1 Department of Radiology, University of Cincinnati, 3188 Bellevue Ave, PO Box 670761, Cincinnati, OH 45267-0761 USA; 2 Department of Nuclear Medicine, University Hospital Zurich, Zurich, Switzerland; 3 Department of Radiology, University of Toronto, Toronto, ON Canada; 4 Heart and Vascular Center, Brigham and Women’s Hospital, Boston, MA USA; 5 Department of Radiology, University of Arizona, Phoenix, AZ USA; 6 Division of Cardiac Surgery, Department of Surgery, University of Cincinnati, Cincinnati, OH USA

**Keywords:** Medical 3D printing, Minimally invasive coronary artery bypass, Half-scaled anatomic model, Pre-surgical planning, Medical additive manufacturing, Likert assessment, Operative room time reduction

## Abstract

**Background:**

Three-dimensional (3D) printing technology has impacted many clinical applications across medicine. However, 3D printing for Minimally Invasive Direct Coronary Artery Bypass (MIDCAB) has not yet been reported in the peer-reviewed literature. The current observational cohort study aimed to evaluate the impact of half scaled (50% scale) 3D printed (3DP) anatomic models in the pre-procedural planning of MIDCAB.

**Methods:**

Retrospective analysis included 12 patients who underwent MIDCAB using 50% scale 3D printing between March and July 2020 (10 males, 2 females). Distances measured from CT scans and 3DP anatomic models were correlated with Operating Room (OR) measurements. The measurements were compared statistically using Tukey’s test. The correspondence between the predicted (3DP & CT) and observed best InterCostal Space (ICS) in the OR was recorded. Likert surveys from the 3D printing registry were provided to the surgeon to assess the utility of the model. The OR time saved by planning the procedure using 3DP anatomic models was estimated subjectively by the cardiothoracic surgeon.

**Results:**

All 12 patients were successfully grafted. The 3DP model predicted the optimal ICS in all cases (100%). The distances measured on the 3DP model corresponded well to the distances measured in the OR. The measurements were significantly different between the CT and 3DP (*p* < 0.05) as well as CT and OR (*p* < 0.05) groups, but not between the 3DP and OR group. The Likert responses suggested high clinical utility of 3D printing. The mean subjectively estimated OR time saved was 40 min.

**Conclusion:**

The 50% scaled 3DP anatomic models demonstrated high utility for MIDCAB and saved OR time while being resource efficient. The subjective benefits over routine care that used 3D visualization for surgical planning warrants further investigation.

**Supplementary Information:**

The online version contains supplementary material available at 10.1186/s41205-024-00222-1.

## Background

MIDCAB of the Left Internal Mammary Artery (LIMA) on the Left Anterior Descending (LAD) [1, 2] coronary artery for patients with isolated proximal LAD stenosis avoids sternotomy and cardiopulmonary bypass. It enables faster recovery, has fewer significant wound infections, and results in less bleeding than traditional bypass grafting [3]. For isolated LAD lesions, postoperative mortality and morbidity is low, and there is excellent short- and long-term survival and freedom from major adverse events and angina [4]. MIDCAB often uses an antero-lateral mini-thoracotomy in the fourth or fifth left ICS. The overall decrease in surgical exposure when compared to more invasive surgeries places greater emphasis on the pre-operative spatial relationships.

3D visualization, defined as the collective methods (including volume rendering and multiplanar reformations) to view a 3D volume on a computer screen [5], from CT data can allow for understanding of the important MIDCAB spatial relationships. While electron beam CT has been entirely replaced by modern CT scanners for cardiovascular care, it was used as early as 1998 for MIDCAB planning to determine the best ICS and to identify the lateral (left–right) distance between the landing point of the LAD and the LIMA [6]. Additional reporting [7] showed that CT could identify those patients with anatomic relationships amenable for MIDCAB, and CT was proven effective to define additional data such as LAD calcification and intramyocardial segments.

To our knowledge, there are no prior peer-reviewed reports demonstrating the use of 3DP anatomic models for patient-specific MIDCAB pre-surgical planning, even though CT data for MIDCAB planning is amenable for printing. In hospital 3DP [5, 8] is increasingly used for surgical planning and other complex cardiovascular interventions [9]. 3DP anatomic models have a high utility in providing the surgeon tactile, volumetric appreciation of the spatial relationships before the procedure. The large majority of 3DP anatomic models are 1:1 scale [10], and when considering the heart alone or the heart and a portion of the aorta, the entire Region of Interest (ROI) can be 3DP for the majority of patients with a desktop machine (6″ × 6″ × 7″ build volume). However, to print the sternum and left ribs, LIMA, heart, and LAD at 1:1 scale, a larger build-tray is required, making desktop monolithic 3DP [11] impossible. The purpose of this project is to describe the use of half-scale anatomic models for MIDCAB planning and to test the hypothesis that pre-operative measurements made from the 3DP anatomic models correlated with those made from the CT images and reference standard measurements made in the OR.

## Methods

### Patients and follow-up

The IRB at the University of Cincinnati College of Medicine reviewed and exempted this retrospective observational study; written informed consent was waived. All patients who underwent MIDCAB between March 2020 and July 2020 were reviewed. The OR time reduction and Likert responses were as reported in a 3DP registry [12, 13]. The patients in this cohort received follow-up for 6 months after the procedure, including an office visit with the attending cardiothoracic surgeon and a review of the electronic medical record.

### CT acquisition

Patients underwent pre-operative prospectively ECG-gated (single phase, 75% R-R interval) CT using two contiguous axial volumes (256 × 0.625 detector rows, 350 ms gantry rotation, GE Revolution, GE Healthcare, Illinois, USA) after intravenous administration (bolus tracking on proximal descending aorta) of 100 mL iodinated contrast material (iohexol 350 mg/mL, GE Healthcare). Neither beta-blockers nor nitroglycerin was administered. Imaging was performed with the patients’ arms at their sides so the CT table would best approach the OR table for spatial relationships. Patients were carefully instructed to take a shallow breath before image data acquisition, and then to stop breathing for the duration of the image data acquisition. DICOM images were reconstructed at 0.625 mm increments for 3D visualization and 3DP.

### Clinical CT reporting and 3D visualization

**Fig. 1:**
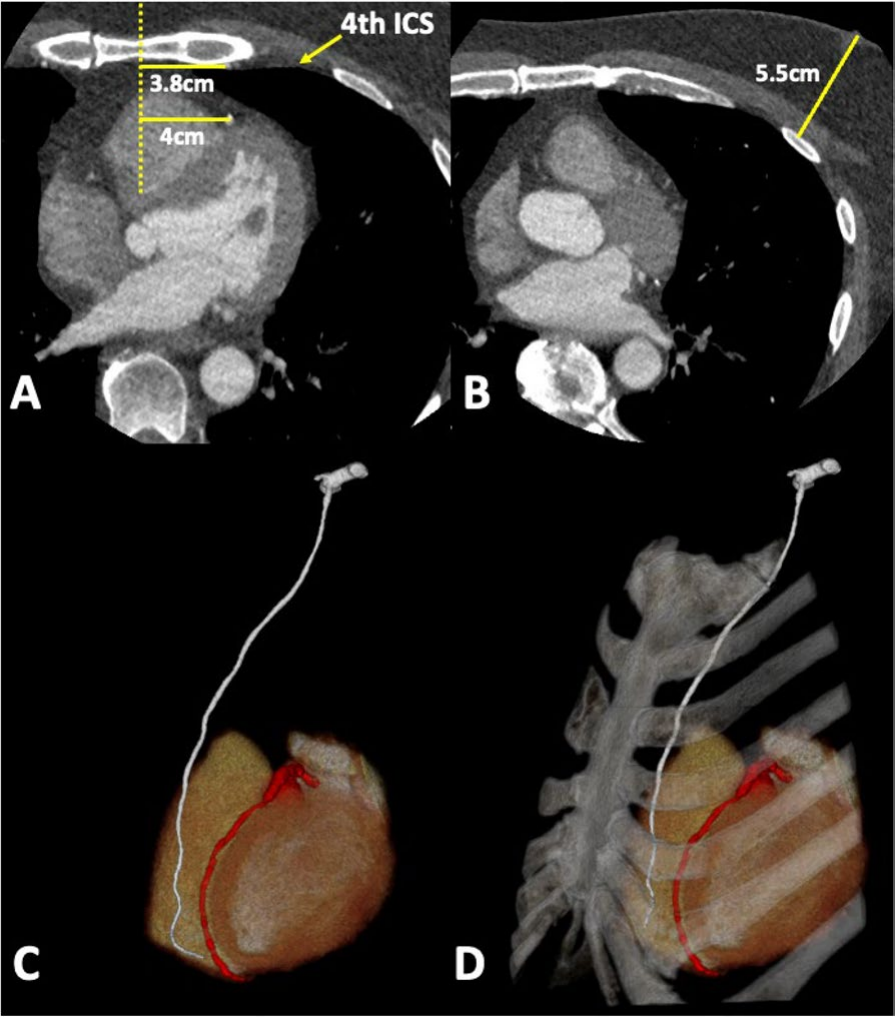
**A** Axial CT images for patient 9 showing the LIMA to MS (3.8 cm) and LAD to MS (4.0 cm) distances in the 4th ICS, and (**B**) full field of view axial image at the level of the right nipple showing the NTR (5.5 cm) distance. **C** Volume rendered image including the LIMA (white) and LAD (red); this is part of the standard 3D visualization, (**D**) The same view of the volume in (**C**) including the bony structures for reference. Because the 3D visualization includes individually segmented parts, the display on a 2D screen can be manipulated by the cardiovascular imager and the surgeon

Standard MIDCAB planning includes inspection and measurements on axial images, plus 3D visualization (Fig. 1). 3D visualization includes MPR images and volume rendering from the thin-section DICOM images using a post-processing workstation launched within the hospital Picture Archiving and Communication System (PACS).

### MIDCAB 3D printing (3DP)

For all patients, a 50% scale anatomic model was 3DP using desktop inverted vat polymerization (Form 3, Formlabs, Massachusetts, USA) with Clear Resin (Supplementary Material: Figure S1) to include 5 anatomic parts: the left first through sixth ribs with the sternum including the manubrium and the xyphoid process, the LIMA, the LAD, the heart, and a marker for the left nipple position on the skin (an important landmark for male patients).

### Objective measurements and determinations

**Fig. 2:**
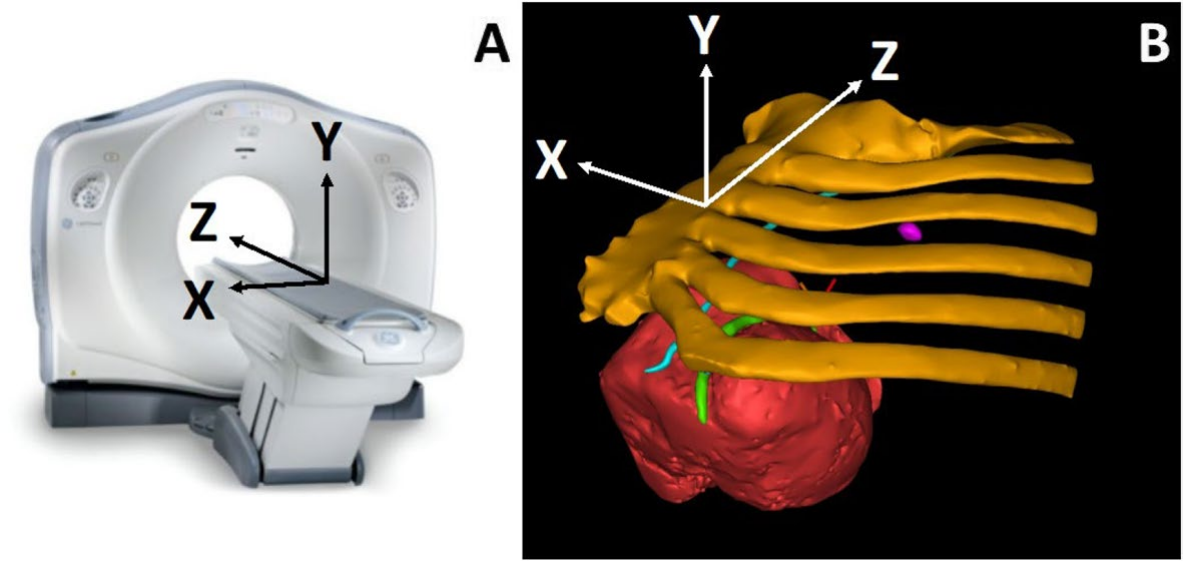
Cartesian coordinate system for distance measurements made from anatomic volumes. **A** The bed of the CT scanner and the OR are considered identical for the measurements with the positive “Z-axis” defined as pointing towards the patient’s head. The X and Y axis were then determined by the right-hand rule for spatial relationships by respect to the CT scanner or the operating room table and (**B**) along the orientation of the anatomic parts segmented for 3DP. Volume rendering after image segmentation shows the same orientation of the cartesian coordinates. Since all the measurements are differences, a specific definition of the origin (x, y, z = 0, 0, 0) was not necessary

Distance measurements were defined according to a Cartesian coordinate system (Fig. 2).

#### Midsternum (MS) to LAD distance

**Fig. 3:**
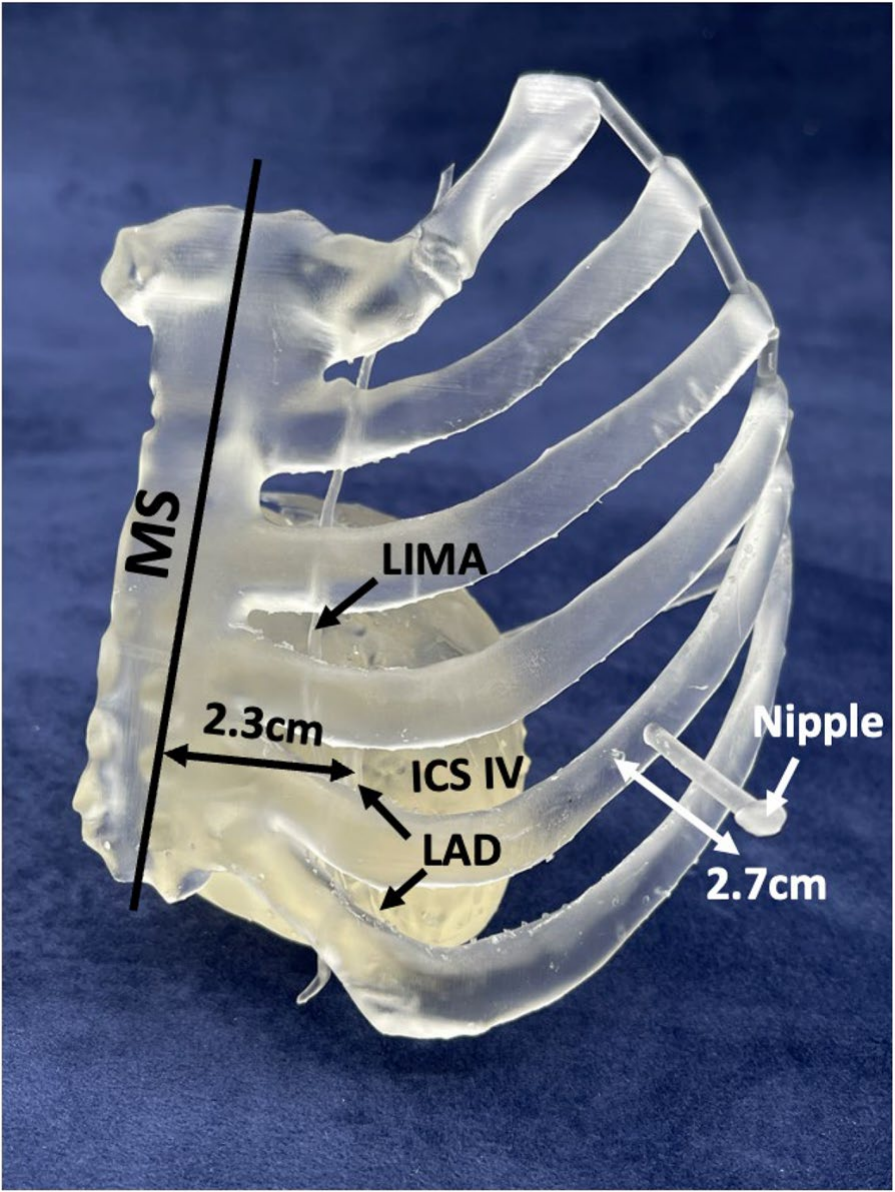
Illustration of length measurements obtained from the 3DP model for patient 9. The LAD is obscured by the left 5th rib as it moves laterally. The MS to LAD distance was determined in the 4th (V) intercostal space (ICS), as this was pre-operatively determined to be the best approach. The measurement of 2.3 cm was on the 50% scaled 3DP model (actual doubled measurement was 4.6 cm). The measurement of the NTR distance, in this case rib 5 was 2.7 cm on the 50% scaled 3DP model (actual doubled measurement was 5.4 cm)

This length measurement was performed in the X (left–right) dimension (Figs. 2 and 3) and included 2 significant figures. Measurements from the model were doubled, based on the 50% scale model. The position on the Z-axis (craniocaudal) for the measurement on the model (Z_model_) and CT (Z_CT_) was determined by the surgeon preoperatively. Z_OR_ was determined on the skin in the OR pre-incision. It was based on anatomic landmarks and the information provided by the model. The position on the Y-axis from the CT (Y_CT_) was taken from the anticipated landing zone preoperatively. Y_model_ was determined from the anterior surface of the sternum on model; and Y_OR_ was determined pre-incision from the patient’s skin. For determining the mid-sternum to LAD distance (along X), all values of Z and Y were presumed to be equal. The CT measurement was performed on the axial plane (Fig. 1A) using the digital measurement function in PACS as well as in Multi-Planar Reformat (MPR). The 3DP and OR measurements were performed with a ruler (1 mm resolution).

#### Nipple to rib (NTR) distance (male patients)

Measurements were made on the 3DP model (and doubled, based on scale), and compared to those taken in the OR and using CT (Axial and MPR). All measurements were made to two significant figures and reflected the shortest distance (independent of the XYZ position) from the NTR.

#### Best ICS

The best ICS was determined by the surgeon and cardiovascular imager from the model preoperatively, and the choice was subsequently correlated with the space as determined from OR findings.

### Subjective evaluation and likert assessments

For each patient, the surgeon and cardiovascular imager preoperatively evaluated the CT and 3DP model to determine the best position on the skin to enter the chest. Considerations included the best surgical exposure and the ability to avoid extension of the mini thoracotomy. The physicians also considered the most favorable approach angle to the intended landing position on the LAD. The time saved by a small precise incision that avoided unnecessary tissue damage caused by extending the incision, changing orientation, and handling the heart so as to make it closer to the surgeon was also considered. For each patient, a “worst case” scenario was considered, defined as the need to convert the procedure to a standard sternotomy with single vessel bypass.

The seven Likert questions from the 3DP Registry [12] were answered by the attending cardiothoracic surgeon post procedure.

The estimated time saved in the OR because of using the 3DP anatomic model in the planning was subjectively recorded for each case by the attending cardiothoracic surgeon.

### Statistical analysis

The MS-LAD and NTR measurement distances from CT, 3DP, and OR were compared using Tukey’s test with *p* < 0.05 considered a statistically significant difference.

## Results

### Patients and follow-up

All 12 patients (Table 1) within the study period underwent MIDCAB with one attending surgeon without any exclusions. The MS to LAD measurements and best ICS was determined for all 12 patients and NTR measurements for all 10 male patients. Three patients underwent MIDCAB for a second graft procedure. All patients were successfully grafted.

**Table 1:** Patient baseline characteristics

No. patients	12
Age, years	63.8 ± 12.2
Male	10 (83%)
Diabetes mellitus	4 (33%)
Hypertension	12 (100%)
Hyperlipidemia	10 (83%)
Smoker	5 (42%)
BMI, kg/m^2^	27.2 ± 3.1

*Abbreviation*: *BMI* Body Mass Index

There were no complications related to either the medical imaging or the 3D printing. One patient (patient 11) required a conversion to sternotomy based on intraoperative findings not related to the anatomic model. This patient required LIMA elongation with a short segment of the great saphenous vein in I-conduit fashion. All patients were discharged home at a median time of 5 days. No patient developed an infection, and no patient required post-operative antibiotics. Regarding the 6-month clinical follow-up with the attending surgeon (one patient with pericardial effusion, two patients with transient acute kidney injury, and one patient with asymptomatic short-term reported ventricular tachycardia), there were no deaths, and no patient had any complication leading to lasting disability or organ failure.

### Clinical 3D visualization

All 12 patients underwent CT acquisition (Fig. 1A, B) without complication and with minimal (if any) imaging artifact. Contrast enhancement was adequate to identify all anatomic parts using MPR images and volume rendering (Fig. 1C-D).

### MIDCAB 3D printing (3DP)

All 12 patients underwent successful 3DP of all anatomic parts at 50% scale (Supplementary Material). 3DP models were evaluated by the surgeon and cardiovascular imager by consensus.

### Objective measurements and determinations

The mean MS-LAD distances were 5.9 ± 2.3 (CT Axial), 6.4 ± 2.1 (CT MPR), 6.4 ± 2.1 (3DP), and 6.5 ± 2.2 (OR). The mean NTR distances were 4.3 ± 1.3 (CT Axial), 4.3 ± 1.3 (CT MPR), 4.3 ± 1.4 (3DP), and 4.4 ± 1.3 (OR). The 3DP model predicted the best ICS used in the OR in all patients (Table 2). The mean procedure time was 326 ± 75 min.

**Table 2:** Linear measurements, best intercostal space, and estimated time saved in the operating room

Patient	CT MS-LAD AXIAL (cm)	CT MS-LAD MPR (cm)	3DP MS-LAD^b^ (cm)	OR MS-LAD (cm)	CT NTR AXIAL (cm)	CT NTR MPR (cm)	3DP NTR (cm)	OR NTR (cm)	Best ICS			Sub. Est. OR time saved (min)
									CT	3DP	OR	
1	5	5.2	5.5	5.8	2.1	2	2	2	IV	IV	IV	30
2	4.8	5.5	5.8	6	4.5	4.3	4	4	V	V	V	50
3	4.6	4.5	4.6	4.6	2.9	2.9	3	3	V	V	V	50
4	5.5	6	6	6	6.2	6.1	6	6	IV	IV	IV	50
5	6.3	6.4	6.6	6.6	4.4	4.5	4.4	4.6	IV	IV	IV	30
6^a^	4.4	4.6	4.8	4.8					IV	IV	IV	30
7	6	6.1	6	6.2	4	4	4	4	V	V	V	50
8	9	8.9	9	9	5.3	5.2	5.6	5.6	IV	IV	IV	30
9	4	4.6	4.6	4.5	5.5	5.6	5.4	5.5	IV	IV	IV	30
10	5.7	7.2	7.5	7.6	5.2	5.2	5.6	5.5	V	V	V	50
11^a^	11.6	11.4	11.6	11.8					IV	IV	IV	50
12	3.6	4.4	4.4	4.5	3	3.1	3	3.4	IV	IV	IV	30

*Abbreviations*: *3DP* 3D Printed, *ICS* Intercostal Space, *LAD* Left Anterior Descending, *MS* Mid-Sternum, *NTR* Nipple to Rib Distance, *OR* Operating Room, *MPR* Multi-Planar Reformat

^a^female patient

^b^doubled measurement, since anatomic models were 3D printed at 50% scale

### Subjective evaluation and likert assessments

The spatial relationship between the LIMA, ribs and sternum, ICS, and nipple was precisely illustrated in the 50% scaled 3DP models (Fig. 3). For all patients, the surgeon reported that the 3DP model provided subjective, improved intuitive understanding of the anatomical relationships when compared to 3D visualization using CT data. Tactile feedback regarding the geometric positions was considered highly valuable. The 3DP models added confidence and pre-procedural planning of the best ICS, and the physical models provided a subjective, improved pre-surgical mapping of the distances measured. The ability to physically hold the printed model and rotate it to view the anatomical relationships from multiple perspectives provided a better sense of the surgical complexity.

The attending cardiothoracic surgeon’s Likert responses were highly positive with the responses falling in either the agree (4) or strongly agree (5) categories for all 7 questions across the 12 patients (Table 3). The average response score across all the patients and questions was 4.5 ± 0.5. When averaged by the 7 questions, the attending cardiothoracic surgeon’s response on Question 7 (after using the 3DP model, I was confident in the treatment plan) had the highest score of 4.9 ± 0.3. The mean score on Question 3 (as a result of using the 3DP model, the treatment plan was altered or refined) was 4.7 ± 0.5.

**Table 3:** Post-procedural Likert responses from the attending cardiothoracic surgeon. The responses are converted into scores as follows: Strong Agree – 5, Agree – 4, Neutral – 3, Disagree – 2, Strongly Disagree – 1

Patients	Likert questions and scores						
	Q1	Q2	Q3	Q4	Q5	Q6	Q7
Pt. 1	5	5	5	5	5	4	5
Pt. 2	4	4	4	4	5	5	5
Pt. 3	4	5	5	4	4	4	5
Pt. 4	4	4	5	4	4	5	5
Pt. 5	4	5	5	5	4	4	5
Pt. 6	4	5	5	5	4	4	5
Pt. 7	4	5	4	4	5	5	5
Pt. 8	4	5	5	5	4	4	5
Pt. 9	4	5	4	5	5	5	4
Pt. 10	4	4	5	5	4	4	5
Pt. 11	4	5	4	5	5	4	5
Pt. 12	4	5	5	4	5	4	5
Mean	4.1	4.8	4.7	4.6	4.5	4.3	4.9
Std. Dev	0.3	0.5	0.5	0.5	0.5	0.5	0.3

Q1. The 3DP model or guide was easy for me to use

Q2. Use of the 3DP model or guide was compatible with other aspects of my approach to this case

Q3. As a result of using the 3DP model, the treatment plan was altered or refined

Q4. Use of the 3DP model or guide was important in this case

Q5. The quality of the 3DP model or guide was adequate

Q6. Before using the 3DP model, I was confident in the treatment plan

Q7. After using the 3DP model, I was confident in the treatment plan

The mean time saved in the OR because of the 3DP anatomic model was subjectively estimated to be 40 ± 10 min (Table 2), which is 11% of the mean procedural time.

### Statistical analysis

**Fig. 4:**
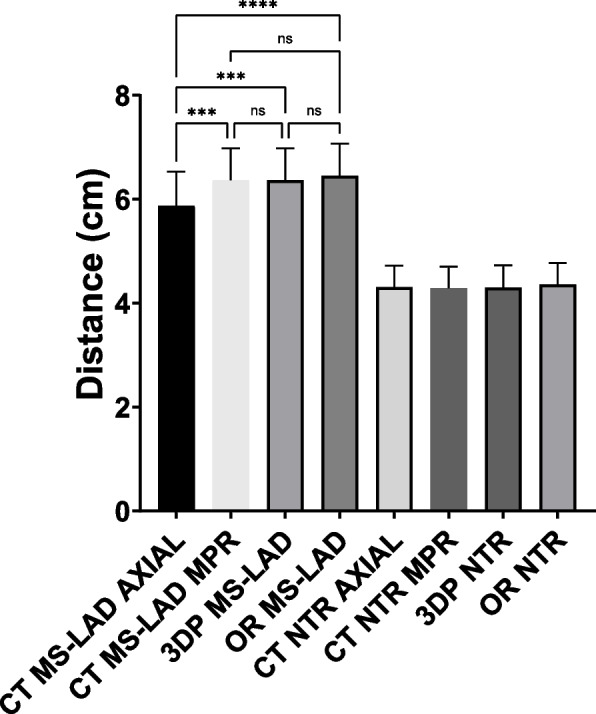
Mean and standard deviation of the MS-LAD and NTR measurements made using CT, 3DP, and in the OR. The CT measurements were recorded in the Axial and MPR orientations. The MS-LAD distances were significantly different between the CT Axial and CT MPR, CT Axial and 3DP, as well as the CT Axial and OR, but not between the CT MPR, 3DP, and OR groups. None of the NTR mean distances were significantly different from each other

The MS-LAD distance was significantly different between the CT Axial and CT MPR (*p* = 0.0006), CT Axial and 3DP (*p* = 0.0006) as well as between the CT Axial and OR group (*p* < 0.0001), but not between the CT MPR, 3DP and OR groups (Fig. 4). The NTR distance was not significantly different between the CT, 3DP, and OR groups.

## Discussion

This project illustrates the accuracy of 3DP anatomic models compared to OR measurements and their high utility for planning MIDCAB surgery. To our knowledge, this report is the first comprehensive study of 3DP anatomic models for MIDCAB planning.

The measurements made on the anatomic model had good agreement with intraoperative measurements, and there was improved confidence in the communication between the cardiovascular imager and the surgeon. The MS-LAD distance measurement was significantly different between the CT Axial and CT MPR, CT Axial and 3DP, as well as between the CT Axial and OR, but not between the CT MPR, 3DP and OR groups (Fig. 4). The likely explanation is that the fact that the MPR, OR and 3DP measurements are in true 3D space at the same Y location (Figs. 2 and 3), whereas the CT measurement is performed in 2D space (axial plane; Fig. 1A) at a slightly different Y location. The NTR distance measurement was not significantly different between the CT (Axial and MPR), 3DP, and OR groups. Although the 3DP and OR measurements are still true 3D measurements compared to the CT (axial plane) measurement, the XYZ location between the measurements is the same for measurement purposes. However, the 3DP and OR measurements of the MS-LAD distance must be interpreted with caution because those measurements are impacted by perspective given the relatively large distance between the location of the measurement ruler and the target site.

The literature reports 3DP using desktop inverted vat polymerization to be accurate within 1 mm, although those data were reported with anatomic phantoms [11]. The current data supports those findings, even at 50% scale, and suggests that 3DP added qualitative information for MIDCAB planning. In our experience, the complexity of the procedure was anticipated knowing the distance between the LAD and the MS (need for LIMA elongation to reach the LAD), and how the surgical exposure could be unfavorable secondary to the surgical depth (NTR distance in males). 3DP was also able to predict in all cases the best ICS to be entered, allowing the surgeon to benefit from improved surgical exposure with a smaller surgical incision. Subjectively, these spatial relationships were best understood with a 3DP model. The 40-min time reduction in the OR across the 12 patients as subjectively estimated by the attending cardiothoracic surgeon suggests that the 3DP anatomic model provides valuable insight into the patient-specific anatomic complexities. Otherwise, these spatial relationships would be first developed in the OR. This is supported by the high mean Likert score assessing the attending cardiothoracic surgeon’s confidence after using the 3DP anatomic model. For the one patient that required a conversion to sternotomy, this additional step was predicted by the considerably longer MS-LAD distance (11.8 cm, when compared to the mean distance of 6.5 ± 2.2 cm across the 12-patient cohort in the OR). In one patient, an intramyocardial LAD was identified proximal to the landing point (Supplementary Material: Figure S2).

This project is also novel because it uses desktop half-scale models for cardiac surgical planning. The most well-cited guidance document suggests that 1:1 anatomic models be 3DP with consultation when it is not possible to print a full-scale model [10]. For structural heart indications, full-scale (1:1) models are essential, for example in test-fitting an occlusion device into the left atrial appendage [14]. Because MIDCAB planning includes the sternum and left ribcage, the ROI is typically too large for desktop printing, the printing hardware used in most in-hospital labs. We printed the words “50% scale” on the model itself, along with the patient medical record number (Supplementary Material: Figure S3A and Figure S4C). We acknowledge that additional research is required to formally evaluate 3DP anatomic models that are decreased in scale from the patient’s anatomy. The tradeoffs for printing a full-scale replica of patient-specific anatomy include costs (see Supplementary Material: 3D Printing) and accessibility to 3D printers with large build volumes.

We acknowledge several additional study limitations, including the small number of patients and the data was collected from a single medical center. MIDCAB was a new procedure at our institution, and we originally planned to develop a control and test group to compare 3D visualization versus 3DP. Based on the subjective benefit of the spatial relationships in the anatomic model, we did not create a control group. This has also been observed among congenital heart disease patients who require 3DP before surgery [15].

Anecdotally, the anatomic models were excellent learning tools for our surgical residents, and were highly appreciated by the patients, who better understood the proposed procedure, increasing their compliance. Overall, the data supported - and we believe that 3DP anatomic models offered the valuable benefit of predicting surgical complexity for MIDCAB. The surgeon better appreciated the anatomy, and there was more confidence towards the minimally invasive coronary revascularization technique because of the expectation of the pertinent interoperative distances.

## Conclusions

The half scaled 3DP anatomic models produced using desktop 3D printing were dimensionally accurate compared to imaging and OR measurements. The models demonstrated high utility for MIDCAB and subjectively saved OR time while being resource efficient. The subjective benefits over routine care that used 3D visualization for surgical planning warrants further investigation.

### Supplementary Information

Supplementary Material 1. 3D Printing (3DP) Appendix. Supplemental Methods: Image Segmentation. Supplemental Methods: Additional Post-Processing. Supplemental Methods: 3D Printing (3DP). Supplemental Results.

## Data Availability

All data generated or analyzed during the study are included in the published article and its supplementary information files.

## Abbreviations

3DP: 3D Printing
MIDCAB: Minimally Invasive Direct Coronary Artery Bypass
LIMA: Left Internal Mammary Artery
LAD: Left Anterior Descending Artery
NTR: Nipple to Rib distance
ROI: Region of Interest
ICS: Intercostal Space
MPR: Multi-Planar Reformatted
MS: Mid-Sternum
OR: Operating Room
PACS: Picture Archiving and Communication System
