# Supplementary material for: Desktop 3D printed anatomic models for minimally invasive direct coronary artery bypass
Source: 3D Print Med. 2024 Jun 12;10:19. doi: 10.1186/s41205-024-00222-1 (PMC11167900; doi:10.1186/s41205-024-00222-1)
Supplement: Supplementary file 1 — Supplementary Material 1. 3D Printing (3DP) Appendix. Supplemental Methods: Image Segmentation. Supplemental Methods: Additional Post-Processing. Supplemental Methods: 3D Printing (3DP). Supplemental Results. [file 41205_2024_222_MOESM1_ESM.docx]

**Supplementary Material: 3D Printing (3DP) Appendix**

Supplemental Methods: Image Segmentation

Supplemental Methods: Additional Post-Processing

Supplemental Methods: 3D Printing (3DP)

Supplemental Results

**Supplemental Methods: Image Segmentation**

The Computed Tomography (CT) Digital Imaging and Communications in Medicine (DICOM) image sets were segmented using Materialise InPrint 3.0 (Materialise, Lueven, Belgium). Patient 9 is illustrated. From the uploaded data (true coronal, axial, and sagittal planes; Figure S1A), the bone thresholding preset was applied to segment the ribs and sternum (Figure S1B). Removal of unwanted anatomy (right hemithorax, plus left lung, spine, clavicle) used a Region-of-Interest (ROI) window resizing along the superior-inferior, anterior-posterior, and lateral directions (Figure S1C), after which a Split Mask operation (Figure S1D) was performed. Final cuts were performed on the segmented volume using a “lasso tool” (not shown) to trim the remaining ribs on the right and the latero-posterior segments of the ribs on the left. The Left Internal Mammary Artery (LIMA) was isolated from the bone and segmented as a unique anatomic part using thresholding, followed by direct planimetry. The Left Anterior Descending (LAD) was segmented as a unique anatomic part by region-growing and direct planimetry. The LIMA and LAD were expanded to a diameter of 5mm – this step deviated from the true diameter but was required for 3DP at 50% scale using desktop inverted Vat Photopolymerization (VP).

A small volume of the myocardium in the interventricular groove directly underneath the LAD was removed by direct planimetry to better highlight the anatomy of the LAD. In male patients the nipple was included. All anatomic parts were modified as needed and verified by an experienced cardiovascular imager both as DICOM overlays and as STL (standard tessellation language) as part of the laboratory quality control.


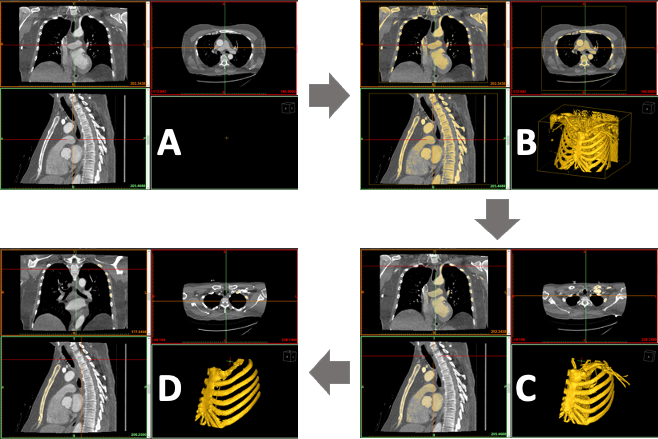


Figure S1. (A) Volume imported to Materialise InPrint 3.0 illustrating true coronal, axial, and sagittal planes. Bottom right panel is initially empty since no volume is segmented. (B) Bone thresholding preset is applied to segment the anatomic parts of interest including the ribs one through six on the left, the sternum, and the manubrium. Additional anatomy is inadvertently included. (C) Resizing the Region-of-Interest (ROI) window superior-inferiorly, anterior-posteriorly, and laterally excludes most extraneous structures. (D) A final Split Mask operation excluded the left clavicle and left subclavian artery with the resulting volume to include the left ribs 1–6 on the left, the sternum, and the manubrium.

The anatomic parts segmented in the final virtual model included the first through sixth ribs with Intercostal Spaces (ICS) on the left, the sternum including the manubrium, the LIMA, the LAD, and a marker for the left nipple in male patients (Figure S2A and Figure S2B). Short segment intramyocardial LAD was noted in the virtual 3D model (Figure S2C). The finalized segmented parts were converted to STL files (Figure S2B) and final post-processing was performed. Additional quality control was performed to ensure that the anatomic parts corresponded to the CT data.

Figure S2. (A) Color coded masks of segmented anatomical parts are overlaid on axial CT image. (B) 3D virtual color-coded model of the segmented anatomy with arrowheads – bone in orange, LIMA in cyan, LAD in bright green, nipple in magenta, and heart in red. (C) Short intramyocardial course of the LAD shown with green arrowhead.

**Supplemental Methods: Additional Post-Processing**

Generated STL files were isolated to remove any floating elements. A smoothing operating was performed followed by wrapping with a 0.5-1mm size mesh to ensure a high quality and uniformly triangulated surface in each model using Materialise 3-matic Medical 14.0 (Materialise, Lueven, Belgium). Any mesh errors were fixed at this stage. The final STL files were re-imported into InPrint 3.0. Connecting pins were manually added between the heart and sternum as well as the heart and the 6th rib (15mm diameter), between adjacent ribs at the free tips (5mm diameter) and between the LAD and myocardium (2-3mm diameter) to ensure adequate support and to enable anatomically accurate monolithic printing (Figure S3A-B). The posterior quarter of the heart including the descending aorta a portion of the left atrium were excluded using a planar cut to decrease material consumption (Figure S3B). Although the LIMA was attached to the ribs at several locations in the merged STL, unattached locations were manually reinforced with 2-3mm diameter connecting pins (not shown) for successful monolithic 3DP. The diameters for the connecting pins were intentionally chosen to be large keeping in mind the subsequent 50% scaling operation. The Medical Record Number (MRN) or an internal institutional case number and “50% scale” labels were stamped onto the sternum and manubrium of the smoothed model before exporting the STL for 3DP (Figure S3A). A final mesh check was conducted within InPrint 3.0 to ensure that the STLs were water-tight and free of errors as part of the laboratory quality control measures.

Figure S3. (A) Antero-lateral view and (B) left-posterior view of the merged virtual 3D model. The connecting pins (white arrowheads) between ribs 1 to 6, the stamped internal institutional case number and model scale information are seen in (A). The 15mm diameter connecting pins between the heart and ribcage as well as the heart and rib 6 along with the 5mm diameter connecting pin between the nipple and rib 5 are highlighted in (B). The planar cut to remove the posterior quarter of the heart is seen in (B).

**Supplemental Methods: 3D Printing (3DP)**

The completed STL file was imported into 3DP preparation software PreForm (Formlabs, Massachusetts, USA) and a scaling factor of 0.5 was applied (Figure S4). The model was manually oriented to eliminate or minimize support scaffolding between the LAD and ribs as well as the heart and LIMA to prevent damage to the LAD and LIMA during support removal (Figure S4A-B). The automatically generated orientation in PreForm was frequently suboptimal for ensuring reduced support scaffolding in this critical area. This is because the software by default optimizes the orientation based on minimizing the largest cross-sectional area parallel to the build surface for reducing peel related forces that are present after every layer, but the algorithm does not factor in specific model regions that maybe more vulnerable to print failure and/or damage during support removal. The region between the sternum and myocardium was also hard to access to remove supports after 3DP, but our manually chosen orientation eliminated support scaffolding in this hard to access region by minimizing the surface area exposed along the print bed. Half scale models were printed with all anatomic parts in one piece using Clear resin (Figure S4C-D) and a Form 3 printer (Formlabs, Massachusetts, USA). The planar cut was performed posteriorly to optimize material consumption (Figure S4A & Figure S4D). Ensuring that the segmented LAD and LIMA vessels in the virtual model were at minimum 5mm in diameter allowed successful 3DP at half scale, since the vessels were at least 2.5mm in diameter in the printed model (Figure S4C).

The support tip size was reduced to 0.4mm to preserve surface finish in the printed model. The support density was set to 0.70–0.80 to optimize scaffolding since larger support densities did not alter the printing result. The model was removed from the build plate and rinsed in the Form Wash (Formlabs, Massachusetts, USA) station for 20min using 91% isopropyl alcohol (IPA). Support structures were then carefully removed without damaging critical structures, particularly the LAD and LIMA (Figure S4D). After support separation the model was manually rinsed again in IPA for a few seconds to dislodge any remaining support debris. The model was subsequently air dried and then UV cured in the Form Cure station (Formlabs, Massachusetts, USA) for 20-25min at 60–65 degrees Celsius. After cooling to room temperature, the prepared model was delivered to the attending surgeon in clinical consultation with the attending cardiovascular imager. The models were not sterilized or sterilizable due to the printing material used (Formlabs Clear Resin), and this information was clearly communicated to the cardiothoracic surgeon. The consultations before and after 3DP were performed in the office environment, and it was noted that models were not taken to the operating room as part of this study.

The 50% scale 3DP models underwent a final quality control step performed by the staff cardiovascular imager who supervises the in-hospital lab and assumes responsibility for the anatomic model. The 3DP model was weighed after removal of support structures using a precision weighing scale. The mean print time was obtained from the print preparation software PreForm, and included the time taken to print the model, support structures and the raft. The resin volume consumption was also obtained from PreForm and included the model, support structures and raft.


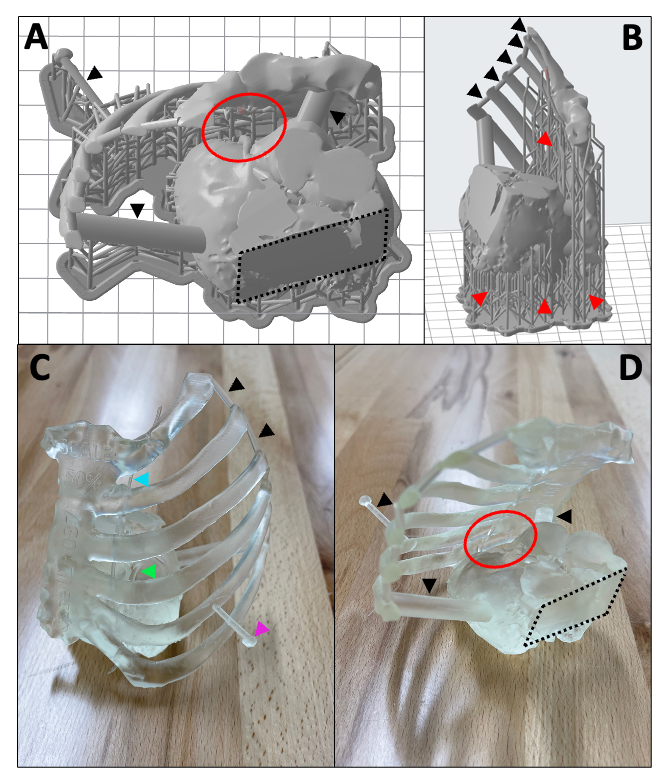


Figure S4. (A) Top view of the model with support scaffolding in PreForm print preparation software with connecting pins called out using black arrowheads. The critical printing region with both the LIMA and LAD vessels is highlighted using a red oval. The dashed planar cutting plane is seen posteriorly. (B) Right side view of the model with the areas of support scaffolding highlighted using red arrowheads and connecting pins between adjacent ribs highlighted using black arrowheads. (C) Antero-superior view of the cleaned, washed and cured 3DP model with the LIMA (cyan arrowhead), LAD (bright green arrowhead), nipple (magenta arrowhead) and connecting pins (black arrowhead) highlighted. The internal institutional case number and scale information is visible on the sternum and manubrium of the printed model. (D) Postero-superior view of the 3DP model with the intact LIMA and LAD highlighted using the red oval and connecting pins highlighted using black arrowheads. The posterior planar cut is also seen.

**Supplemental Results**

The mean mass of the scaled anatomic models was 125.8 +/- 61.8 grams after removal of supports and raft and all 12 could be held comfortably in one hand. The mean print time was (11 +/- 4 hours). The mean volume of clear resin was 142.6 +/- 70.9 cc, translating to a material cost of $21.25 +/- $10.56, based on a cost of $149 per liter. The volume of resin was estimated to be about 85% less than what would be consumed for a full-scale anatomic model, and the estimated savings in 3DP time was about 19 hours. In essence, the 50% scaled model resulted is roughly 1/8th the material consumption compared to that of a full scaled model since ½ scale along each of the XYZ dimensions translates to 1/8th the original volume. The time required to segment all anatomical structures was 3–4 hours with an additional 1–2 hours in software post-processing time. Cleanup of the models took an additional 1-1.5 hours.

Form 3 printer models are always printed fully dense, and therefore scaling is the primary method to conserve material other than hollowing the internal volume. However, hollowing of the internal volume necessitates internal support structures that are difficult to remove and can negatively affect the quality of anatomical feature visualization using transparent material. Extra precaution was taken during the removal of support structures to maintain the anatomic accuracy of the LAD and LIMA, the most fragile structures in the model. The translucent appearance of Clear Resin was conducive to visualizing the internal and intricate anatomical features present in the printed models.

The removal of roughly a quarter of the posterior portion of the heart did not negatively impact the model efficacy in surgical planning, yet it resulted in additional material savings supplementary to that resulting from the 50% model scaling. The removal of this posterior portion also reduced the cleanup time since the number of support structures and the size of raft were correspondingly reduced. As mentioned, the scaling and posterior heart section cut resulted in a large reduction in total model preparation time. The actual 3DP time is typically the most time intensive step in the workflow and we were able to reduce this to ~ 8–10 hours from ~ 30–40 hours required to 3DP the full 100% scale model in 2 pieces. This helped to 3DP the model in a single overnight print session.
